# A Blindfolded Pediatric Trauma Simulation and Its Effect on Communication and Crisis Resource Management Skills

**DOI:** 10.7759/cureus.19484

**Published:** 2021-11-11

**Authors:** Juan X Lopez de Alda, Nirali Patel, Neil McNinch, Rami A Ahmed

**Affiliations:** 1 Pediatric Emergency Medicine, Akron Children's Hospital, Akron, USA; 2 Pediatric Emergency Medicine, Golisano Children's Hospital of Southwest Florida, Fort Myers, USA; 3 Epidemiology and Public Health, Akron Children's Hospital, Rebecca D. Considine Research Institute, Akron, USA; 4 Emergency Medicine, Indiana University School of Medicine, Indianapolis, USA; 5 Emergency Medicine, Methodist Hospital, Indianapolis, USA

**Keywords:** pediatrics emergency, trauma management, good communication skills, simulation training, stress

## Abstract

Background

Miscommunication is a common cause of medical errors and patient harm. Simulation is a good tool to improve communication skills, but there is little literature on advanced techniques to improve closed loop communication (CLC) in an effort to minimize medical errors. This study looks to evaluate whether blindfolding simulation participants is an effective tool in improving communication, and whether this advanced teaching technique is useful for critical pediatric scenarios.

Methods

Participants included Emergency Medicine (EM) residents and Pediatric EM fellows with Advanced Trauma Life Support (ATLS) certification. Participants were randomized into groups and completed a pediatric trauma scenario. Recorded simulations were reviewed by three independent faculty for primary objective measures of total instances of communication and CLC utilization during critical actions in the simulation. The secondary objective was the perceived stress load by participants when utilizing this teaching methodology. Wilcoxon rank sum test (WRS), Fisher’s exact test (FET), and Cochran-Armitage test (CAT) were utilized for statistical analysis.

Results

Statistically significant differences were noted in total communication between groups. Median and interquartile ranges (IQR) of total instances of communication were 17.0 (14.7-17.1) in non-blindfolded groups versus 21.0 (19.0-22.0) in blindfolded groups (p-value=0.002). Statistically significant increase in CLC was noted during the critical action of monitor placement in the blindfolded group (OR=13.7, 95% CI=1.4-133.8). No differences were noted in crisis resource management (CRM) scores. NASA Task Load Index (NASA-TLX) scores of both groups revealed similar stress levels. Statistical testing based upon the year of training was limited by small sample size and large number of categories.

Conclusions

Blindfolded simulations increased total instances of communication overall and improved CLC in one critical action without increasing stress levels. The blindfolded trauma simulation exercise is an effective advanced technique to reinforce CLC utilization and communication skills.

## Introduction

Communication errors remain a leading cause of preventable medical errors and patient harm in medicine, and are considered the root cause for one-third of all sentinel events reported to the Joint Commission [1-3]. These errors are more likely to occur during medical resuscitations or traumas due to the challenges in communication within a multidisciplinary team, increased stress levels, sensory overload, and the potential for distractions [4]. They are also frequent due to lack of regular training, evaluation, and feedback. Contemporary crew or crisis resource management (CRM) training teaches situational awareness, communication skills, anticipation of errors, and error containment and management strategies in high-intensity scenarios in order to minimize preventable patient harm [5,6]. Closed loop communication (CLC) is regularly taught in acute care certification courses as the optimal and safest method for communicating critical clinical information to minimize medical errors in high-stress situations, and this type of communication can be strengthened through CRM training [7].

Simulation has been shown to be an effective CRM training method to improve leadership, communication, and teamwork skills, but there is little description in the literature of advanced simulation techniques to refine communication in particular [8-9]. A pilot study by Hughes et al. utilized blindfolding during simulations as an advanced teaching technique to improve CLC [10-11]. It accomplished this by removing the visual cues many practitioners rely on, thereby forcing them to utilize and develop their communication skills throughout the exercise. These blindfolded simulation exercises could translate to a reduction in preventable medical errors related to miscommunication by fostering improvement in CLC and overall communication skills.

Previous studies in pediatric emergency department resuscitations have shown that, during post-resuscitation debriefings, communication during the resuscitation is regularly cited as an area of weakness [12]. This is likely compounded by the fact that pediatric and adult providers do not feel adequately prepared to participate in pediatric resuscitations, pediatric traumas, pediatric arrests, or emergency procedures [13-17]. These providers may benefit from a controlled training methodology, such as simulation, that could facilitate development of resuscitation experience, improve their CRM skills, and help develop high-quality communication habits during pediatric trauma emergencies. This study aims to establish a baseline comparison of CLC orders, communication CRM skills specifically, overall CRM behaviors, and perceived participant stress levels between team leaders in blindfolded pediatric trauma simulation exercises and non-blindfolded pediatric trauma simulation exercises to determine whether this advanced teaching technique will improve comfort with pediatric emergencies while improving communication to minimize preventable medical errors.

## Materials and methods

This was an exploratory, randomized, pilot cohort study of pediatric trauma simulations utilizing a novel training methodology. Akron Children's Hospital IRB issued approval for this study (1166938-1). A single pediatric trauma scenario of moderate to high complexity was constructed prior to subject recruitment and was used uniformly for all participants. The constructed scenario was not based on any actual patient information, and any resemblance to persons living or deceased is a coincidence. Participants included pediatric emergency medicine fellows (n=8) and emergency medicine residents (n=20) that had completed their Advanced Trauma Life Support (ATLS) certification and were recruited on a volunteer basis (n=28) with informed consent. Participants were randomized into groups as blindfolded or non-blindfolded simulation leaders via a random number draw, with odd numbers representing blindfolded scenarios and even numbers representing non-blindfolded scenarios. The study participants were assessed on utilization of CLC orders and total instances of communication. Prior to starting the simulation, each participant was shown a standardized PowerPoint presentation reviewing CLC strategies and then a video demonstrating a sample blindfolded simulation scenario. Upon entering the simulated resuscitation bay, all participants were seated in a chair five feet from the patient’s bed to minimize variance, with those wearing blindfolds facing backwards. Comprising the rest of the trauma team were three embedded participant nurses assigned the roles of airway support, procedure completion, or medication preparation/administration. Nurses were provided the scenario in advance to review and were instructed on appropriate CLC and how to communicate with the leaders during the scenario. A rehearsal simulation was conducted with the scheduled nursing staff at the beginning of each simulation day to acclimate them both to the mannequins, the scenario, and how they were expected to communicate with the participants.

The trauma scenario consisted of pediatric patient presenting in uncompensated hemorrhagic shock secondary to blunt abdominal trauma and an open pelvic fracture. The simulation scenario and critical actions can be found in Appendix 1. Each simulation was 10 minutes in duration followed by a 10-minute debriefing session. After the debriefing, participants were provided a post-participation survey and NASA Task Load Index (NASA-TLX) forms for completion. NASA-TLX is a multi-dimensional tool that assesses perceived workload stress in order to assess team effectiveness. NASA-TLX is scored on a scale of 0-450 for each subcategory, and weighted ratings are on a scale from 0-100, where an optimal level of perceived stress for a training tool is from 40-70. Emergency medicine, pediatric emergency medicine, and simulation faculty members completed CRM forms after each scenario was completed. All simulations were recorded for review and transferred to secured flash drives. Recorded scenarios were reviewed by three independent pediatric emergency medicine faculty for total instances of communication, CLC orders, and completion of critical actions. An appropriately completed CLC order included verbal communication of 1). an initial order; 2). confirmation of the order; 3). completion of the order; and 4). acknowledgement of completion of the order. Each independent faculty reviewer was provided the CLC presentation prior to completing reviews.

Data analysis utilized summary statistics and distributional assessments for continuous data, and frequencies and percentages for categorical data. Wilcoxon rank sum test (WRS), Fisher’s exact test (FET), and Cochran-Armitage test (CAT) were used for statistical analysis. Unless otherwise noted all testing was two-tailed and evaluated at the Type 1 error rate of alpha=0.05 level of statistical significance. An inter-rater reliability assessment was conducted by determining the intraclass correlation coefficient (ICC) for each step (1-4) of CLC individually as well as steps 1-4 combined (a completely closed communication loop) for each critical action as well as overall instances of communication throughout the entire simulation scenario. ICC was 0.92 for step 1, 0.58 for step 2, 0.69 for steps 3 and 4, 0.76 for completion of loop, and 0.71 for overall instances of communication, demonstrating significant variability in scores between raters. For purposes of statistical analyses, when counting the frequency of each step of CLC and number of loop closures, the number value used was the one agreed upon by at least two of the three reviewers, and the average of the numbers provided by the three raters was used for overall instances of communication.

## Results

There were 13 non-blindfolded participants (46%) and 15 blindfolded participants (54%) enrolled in the study. Both blindfolded and non-blindfolded groups were similar in terms of age (CAT: p-value=0.964), years of training (CAT: p-value=0.953), and gender (FET: p-value=0.276). Years of medical training between both groups were similar, with both of them having an even distribution of higher and lower level trainees.

As seen in Table 1, the number of months that participants worked in an adult emergency department was similar between groups (p-value=0.771). The number of months that participants had worked in pediatric rotations was similar between groups as well (p-value=0.557), with over 50% having less than six months of total experience in both groups. This indicates a trend towards more emergency medicine than pediatric experience amongst participants, consistent with a larger number of participants being emergency medicine residents.

**Table 1:** Clinical experience of study cohorts (*Cochran-Armitage Trend Test)

Adult Emergency Medicine Experience			Pediatrics Experience		
Number of Months	Blindfolded n (%)	Non-blindfolded n (%)	Number of Months	Blindfolded n (%)	Non-Blindfolded n (%)
0-5	0 (0)	1 (7.7)	0-3	4 (26.7)	5 (38.5)
6-10	5 (33.3)	3 (23.1)	4-6	5 (33.3)	4 (30.8)
11-15	3 (20.0)	2 (15.4)	7-9	1 (6.7)	1 (7.7)
16-20	2 (13.3)	1 (7.7)	10-12	1 (6.7)	0 (0)
>20	5 (33.3)	6 (46.2)	>12	4 (26.6)	3 (23)
Total	15 (100)	13 (100)	Total	15 (100)	13 (100)
	p-value	0.771*		p-value	0.557*

Regarding prior experience with real-life trauma scenarios, the groups were evenly distributed when it came to participation in (p-value=0.300), and leading (p-value=0.797), adult traumas. As observed in Table 2, over 50% of the participants from both groups had involvement in >20 adult trauma scenarios. There was a difference between groups in history of experience leading adult traumas that was not significant, with under 50% of the blindfolded group having led >16 traumas when compared to under 50% in the non-blindfolded group. When it came to real-life pediatric traumas, the groups were evenly distributed in both history of participation in (p-value=0.688), and leading (p-value=0.849), pediatric traumas. The majority of learners in both groups were inexperienced in leading pediatric traumas, with most (66.7% and 69.2% in the blindfolded and non-blindfolded groups respectively) having been leaders in two or less pediatric traumas throughout their medical training.

**Table 2:** Trauma experience of study cohorts (*Cochran-Armitage Trend Test)

	Adult Trauma Cases Participated In		Adult Trauma Cases Lead	
Number	Blindfolded n (%)	Non-blindfolded n (%)	Blindfolded n (%)	Non-blindfolded n (%)
0-5	2 (13.3)	0 (0)	3 (20.0)	4 (30.8)
6-10	1 (6.7)	1 (7.7)	4 (26.7)	1 (7.7)
11-15	3 (20.0)	2 (15.4)	2 (13.3)	1 (7.7)
16-20	0 (0)	1 (7.7)	2 (13.3)	3 (23.1)
>20	9 (60.0)	9 (69.2)	4 (26.7)	4 (30.8)
Total	15 (100)	13 (100)	15 (100)	13 (100)
	p-value	0.300*	p-value	0.797*
	Pediatric Trauma Cases Participated In		Pediatric Trauma Cases Lead	
Number	Blindfolded n (%)	Non-blindfolded n (%)	Blindfolded n (%)	Non-blindfolded n (%)
0-2	1 (6.7)	1 (7.7)	10 (66.7)	9 (69.2)
3-4	2 (13.3)	3 (23.1)	0 (0)	0 (0)
5-6	4 (26.7)	3 (23.1)	1 (6.7)	0 (0)
7-8	2 (13.3)	1 (7.7)	0 (0)	2 (15.4)
>8	6 (40.0)	5 (38.5)	4 (26.7)	2 (15.4)
Total	15 (100)	13 (100)	15 (100)	13 (100)
	p-value	0.688*	p-value	0.849*

Comparison of communication utilization revealed evidence of a statistically significant difference in total instances of communication between the two groups. Median and interquartile ranges (IQR) of total number of communications were 17.0 (14.7-17.1) in non-blindfolded groups versus 21.0 (19.0-22.0) in blindfolded groups (WRS: p-value=0.002). Although CLC completion was poor in both groups, there were statistically significant differences in CLC during the critical action of placing monitors, with blindfolded groups more likely to close the loop (OR=13.7, 95% CI = 1.4-133.8). During monitor placement, the most commonly missed steps of CLC were steps 3 and 4, with non-blindfolded participants 6.2 (95% CI of OR = 1.2 - 32.0) and 14.6 (95% CI of OR = 2.2 - 97.6) times more likely to fail completing those steps respectively compared to blindfolded groups. No significant differences were noted in CRM scores or NASA TLX scores. NASA TLX in both groups were above the validated cut off of 70 for “too stressful” of an experience (71.7 in the non-blindfolded group, 73.7 in the blindfolded group).

CLC utilization was most consistent in the critical action of assessing and securing the patient’s airway, but it was still only utilized by approximately 60% of the participants. The critical actions with the least completion of CLC were activation of the massive transfusion protocol and disposition to the operating room.

As a secondary, exploratory analysis, when reviewing CRM and NASA TLX scores based on levels of training, the small sample sizes precluded conduction of an inferential statistical analysis. However, evaluation of the summary statistics could yield insight into trends of both CRM and NASA TLX scores that could benefit from further examination in future research.

As observed in Table 3, overall CRM scores trended toward improvement with level of training. There is a drop in overall scores around those with four years of experience before the scores begin to improve with year of training. Leadership, resource utilization, and communication skills also shared this same trend. Problem solving skills improved consistently with more years of training. There appears to be improvement in the majority of the CRM skills categories with increased years of medical training, but we were unable to interpret for statistical significance due to the small sample size. 

**Table 3:** Crisis resource management scores SD = Standard deviation; IQR = Interquartile range.

		Ottawa GRS Score				
	Overall	Leadership	Problem Solving	Situational Awareness	Resource Utilization	Communication
Years of Training	Mean (SD) median (IQR)	Mean (SD) median (IQR)	Mean (SD) median (IQR)	Mean (SD) median (IQR)	Mean (SD) median (IQR)	Mean (SD) median (IQR)
1 (n=3)	3.3 (1.5) 3.0 (2.0 – 5.0)	4.7 (1.2) 4.0 (4.0 – 6.0)	4.7 (1.5) 5.0 (3.0 – 6.0)	4.0 (1.7) 3.0 (3.0 – 6.0)	5.3 (1.5) 5.0 (4.0 – 7.0)	6.0 (1.0) 6.0 (5.0 – 7.0)
2 (n=8)	4.5 (1.3) 5.0 (3.0 – 5.5)	5.0 (0.5) 5.0 (5.0 – 5.0)	5.1 (1.4) 5.5 (4.0 – 6.0)	4.9 (1.0) 4.5 (4.0 – 6.0)	4.9 (1.4) 5.5 (3.5 – 6.0)	5.3 (1.2) 5.0 (5.0 – 6.0)
3 (n=3)	5.7 (1.2) 5.0 (5.0 – 7.0)	6.3 (1.2) 7.0 (5.0 – 7.0)	5.3 (1.5) 5.0 (4.0 – 7.0)	5.3 (1.5) 5.0 (4.0 – 7.0)	6.0 (1.0) 6.0 (5.0 – 7.0)	6.3 (1.2) 7.0 (5.0 – 7.0)
4 (n=9)	5.2 (1.2) 5.0 (5.0 – 6.0)	5.9 (0.9) 6.0 (6.0 – 6.0)	5.6 (0.9) 6.0 (5.0 – 6.0)	5.3 (0.9) 5.0 (5.0 – 6.0)	5.4 (1.1) 5.0 (5.0 – 6.0)	5.7 (0.9) 5.0 (5.0 – 6.0)
5 (n=4)	5.5 (1.7) 6.0 (4.5 – 6.5)	6.3 (1.5) 7.0 (5.5 – 7.0)	6.0 (0.8) 6.0 (5.5 – 6.5)	6.3 (1.0) 6.5 (5.5 – 7.0)	6.5 (0.6) 6.5 (6.0 – 7.0)	6.5 (1.0) 7.0 (6.0 – 7.0)
6 (n=1)	7.0 (-) 7.0 (7.0 – 7.0)	7.0 (-) 7.0 (7.0 – 7.0)	7.0 (-) 7.0 (7.0 – 7.0)	7.0 (-) 7.0 (7.0 – 7.0)	7.0 (-) 7.0 (7.0 – 7.0)	7.0 (-) 7.0 (7.0 – 7.0)

As observed in Table 4, the scores of the overall and weighted stress ratings did not show any consistent trend with years of medical training. Notably those with one, four, and five years of training felt like the simulation was not excessively stressful of an experience, whereas those with two, three, and six years of training did give the simulation experience a score that would make it too stressful to be useful.

**Table 4:** NASA task load index (NASA-TLX) scores SD = Standard deviation; IQR = Interquartile range.

	NASA TLX						
	Weighted Rating	Mental Demand	Physical Demand	Temporal Demand	Performance	Effort	Frustration
Years of Training	Mean (SD) Median (IQR)	Mean (SD) Median (IQR)	Mean (SD) Median (IQR)	Mean (SD) Median (IQR)	Mean (SD) Median (IQR)	Mean (SD) Median (IQR)	Mean (SD) Median (IQR)
1 (n=3)	67.3 (10.2) 72.0 (55.7 – 74.3)	336.7 (132.9) 325.0 (210.0 – 475.0)	0.0 (0.0) 0.0 (0.0 – 0.0)	176.7 (125.0) 180.0 (50.0 – 300.0)	183.3 (123.4) 150.0 (80.0 – 320.0)	130.0 (98.2) 100.0 (50.0 – 240.0)	183.3 (198.6) 140.0 (10.0 – 400.0)
2 (n=8)	71.0 (9.5) 73.8 (66.8 – 76.0)	364.4 (118.9) 400.0 (310.0 -437.5)	0.0 (0.0) 0.0 (0.0 – 0.0)	199.4 (130.1) 210.0 (80.0 – 320.0)	170.0 (76.6) 187.5 (112.5 – 212.5)	117.5 (64.0) 135.0 (55.0 – 160.0)	214.4 (113.4) 267.5 (125.0 – 300.0)
3 (n=3)	76.0 (4.9) 73.7 (72.7 – 81.7)	275.0 (215.0) 340.0 (35.0 – 450.0)	0.0 (0.0) 0.0 (0.0 – 0.0)	183.3 (72.2) 225.0 (100.0 – 225.0)	223.3 (160.6) 240.0 (55.0 – 375.0)	153.3 (77.7) 130.0 (90.0 – 240.0)	305.0 (217.4) 380.0 (60.0 – 475.0)
4 (n=9)	61.2 (9.1) 60.7 (56.3 – 69.7)	375.0 (56.1) 387.5 (335.0 – 412.5)	3.8 (10.6) 0.0 (0.0 – 0.0)	115.6 (65.4) 110.0 (62.5 – 172.5)	140.4 (69.3) 120.0 (100.0 – 150.0)	168.1 (57.2) 140.0 (130.0 – 202.5)	115.6 (153.1) 30.0 (15.0 – 217.5)
5 (n=4)	69.8 (16.1) 75.2 (60.3 – 79.3)	437.5 (52.0) 437.5 (400 – 475.0)	0.0 (0.0) 0.0 (0.0 – 0.0)	273.8 (106.4) 265.0 (187.5 – 360.0)	140.0 (117.3) 152.5 (40.0 – 240.0)	123.8 (36.4) 130.0 (97.5 – 150.0)	72.5 (49.7) 70.0 (37.5 – 107.5)
6 (n=1)	72.7 (-) 72.7 (72.7 – 72.7)	450.0 (-) 450.0 (450.0 – 450.0)	0.0 (-) 0.0 (0.0 – 0.0)	280.0 (-) 280.0 (280.0 – 280.0)	210.0 (-) 210.0 (210.0 – 210.0)	120.0 (-) 120.0 (120.0 – 120.0)	30.0 (-) 30.0 (30.0 – 30.0)

The most consistently demanding TLX subcategory during the simulations was Mental Demand for most groups of years of training. Amongst those participants with three years of training, the most demand and stress came from Frustration, although it is not clear whether this is from frustration with the scenario, with their lack of pediatric experience, or with the blindfolding. The TLX score category that was universally the lowest in this project was that of Physical Demand across all participants regardless of years of training.

As observed in Table 5, post-simulation surveys indicate this was a very well received training exercise, with most participants enjoying the opportunity to improve clinical skills in pediatric resuscitations in a controlled environment and getting experience in a leadership role. Participants appreciated the time spent in the debriefing to help cement the learning points regarding appropriate management of pediatric traumas and how to better communicate with staff in a position of leadership. All the participants that were blindfolded agreed that being blindfolded made the simulation more challenging by removing their regularly relied on visual cues, but still considered it a great learning experience and would volunteer again if they were readily available.

**Table 5:** Post-curriculum evaluation

Free text responses from post-simulation surveys	
Survey questions	Common responses and selected quotes
What was the best part of this training session?	“Practicing a high stress clinical scenario without the stress of a real child decompensating.” “Forcing me to verbalize each step in the process, which will help me to remember each step when I am under more pressure in a real world scenario.” “Blindfold forces the team leader to step back. EM is so procedure driven we get used to hands on at bedsides and sometimes miss the ‘big overall picture.’” “Practice running peds trauma”
What was the most challenging part of this training session?	“Being blindfolded.” “Not being able to directly interact with the patient.” “Limiting visual cues made it very difficult.” Pediatric medication dosing
What part of this training session needs the most improvement?	Try to encourage junior residents to volunteer more The beginning instructions could have been clarified more, especially for the blindfolded group
Other comments	“Honestly, great exercise and well put together.” “Helpful to do a simulation focused on communication, allowed me to recognize importance of it.”

## Discussion

Blindfolding of the team leader increased total instances of communication overall and also improved CLC in the critical action of monitor placement. The most commonly missed steps of CLC in this critical action were verbalizing completion of the order and verbalizing acknowledgment of order completion, with the blindfolded group performing better than the non-blindfolded group. Overall, the blindfold technique did not demonstrate improved CRM scores. Although summary statistics showed a trend toward improved CRM scores based on years of training, the small number of participants per group did not provide enough power to establish statistical significance in this trend.

The results obtained from the study appear to show a trend consistent with our assumption that removing visual cues would increase verbal communication and overall improve communication skills. The measured increase in total instances of communication could theoretically allow more opportunities to improve the frequency of good CLC behaviors if practiced in a controlled training environment. More regular blindfolded simulations could be useful as a teaching modality to reinforce these good behaviors, making them a habit for learners to apply instinctively in real-life critical scenarios. Additionally, performing blindfolded simulations more frequently could potentially decrease the stress levels and challenge for the learners through experience and familiarity, with both the pediatric simulation setting and with having to wear a blindfold, based on the trends seen in summary statistics per year of training.

As seen in Table 1 and Table 2, the majority of learners had extensive adult emergency medicine and trauma leadership experience, but a very small amount of pediatric trauma experience and time spent on pediatric rotations, which is likely attributable to a large number of adult emergency medicine trained learners compared to pediatric emergency medicine trained learners. As management of critical pediatric patients is a known area of discomfort for many adult emergency providers, this may have had an impact on both how scenarios went overall and on the reported participant stress levels. Having had less exposure to pediatric medication weight-based doses, algorithms, and equipment sizes could theoretically cause a cognitive overload that detracted from focus on communication skills and techniques, thereby contributing to the overall poor CLC completion amongst learners. Conducting these simulations with two large cohorts, one of pediatric emergency medicine learners and one of emergency medicine learners, would be beneficial to examine this effect in future studies.

The trend towards improvement in CRM skills with years of medical training could make this a more beneficial teaching method for the more experienced. Their prior experience would facilitate focusing on the communication aspect of CRM specifically rather than being overwhelmed with the other CRM skills also required during the simulations. The majority of participants with more than three years of medical training (n=13) did not find the simulations to be too stressful regardless of whether they were blindfolded or not, presumably due to more familiarity with appropriate critical medical management and confidence in making decisions, supporting the theory that this might be a better teaching method at higher levels of training. 

A larger, more longitudinal study would be beneficial to further examine these findings, and the study could be expanded by providing follow-up simulations at regular intervals to assess for persistent changes in communication behaviors as well as for retention of other CRM skills. This would additionally allow observation of trends in overall stress levels reported with subsequent simulations, helping to differentiate whether high-stress scores may be due to inexperience with pediatric simulation in general or with having to wear a blindfold. Additionally, an expansion of pediatric scenario types, rather than just trauma scenarios, would be ideal to help reduce future medical errors and increase competency in pediatric management across the board.

This study’s largest limitation was its small sample size. Having only 28 participants meant that analysis of the data in terms of years of training was underpowered and statistical significance of results could not be assessed. This also limited the analysis of the primary objectives of CLC completion of critical actions. This study was also limited to a single-center study. Although there were EM residents from five different programs participating, the majority of them came from a single program where the residents do not have regular exposure to pediatric patients, which could skew results due to both inexperience and heightened stress levels in pediatric simulation scenarios. The three independent raters are local clinical faculty members and have worked with the majority of learners, potentially introducing some bias.

Another area of variance was the embedded participant nursing staff, as the same nurses could not participate during every scheduled simulation. As new embedded participant nurses had to be trained and learn the scenario before each day of simulation, there was an increased likelihood of them introducing errors or making the scenario artificially more challenging due to varying levels of experience. A last possible area that could have affected performance of the learners came from an attempt to minimize bias in the study design. All participants were forced to be seated five feet from the patient regardless of whether or not they were blindfolded, and those that did not have a blindfold in place found it stressful to be restricted from approaching the patient or standing up as they normally do in their day to day practice, which could have affected the way they would normally manage a pediatric trauma.

## Conclusions

In this study, blindfolded simulations increased total instances of communication overall in comparison to traditional simulation scenarios and improved CLC in the critical action of monitor placement without significantly increasing perceived stress levels of participants. Although both groups considered the exercise to be too stressful to be a good learning technique, the overall weighted stress scores given were incredibly close to the optimal range for a teaching tool.

## Appendices

Appendix 1

Pediatric Trauma Sim

Case: Rollover motor vehicle accident (MVA)

Abdominal and pelvic injury

Case: 10 mins

Debrief: 10 mins

PATIENT: Marjorie Smith

CC: Rollover MVA, unresponsive, ejected

HPI: Patient is a 7-year-old girl involved in an MVC who presents to the ED by EMS from the scene of the accident. She is not accompanied by any family members. She is minimally responsive and hypotensive, with abrasions all over her body.

EMS: “This is a 7-year-old girl that was involved in an MVC with her family about 20 minutes ago. She was unrestrained and ejected from the car. We found her unresponsive at the scene. She was hypotensive and we have failed all attempts at obtaining IV access. We have placed her in a C-collar and on a backboard. She has abrasions all over her lower extremities and back. VS at the scene were O2 Sat of 92%, BP of 95/60, HR of 145, RR of 28 with poor effort. She has been bagging easily en route. Trauma was activated from the scene.”

WEIGHT for the scenario: 25kg using Broselow (once resident asks for weight)

PMHx:

UNKNOWN

Medication History:

UKNOWN

Medication Allergies:

UKNOWN

SHx:

UNKNOWN                                                                           

FHx:

UNKNOWN

ROS:

Unable to be obtained secondary to change in mental status

PE:

Glasgow Coma Scale: 8

Motor: 4 (flexion/withdrawal to painful stimuli)

Eyes: 1 (does not open eyes)

Verbal: 3 (utters inappropriate words/moans)

General: Minimally responsive female. 

HEENT: NC/AT, PERRL, 3 mm. Midface stable. No battle/raccoon sign. C-collar in place. No cervical spine step off/deformity. No anterior neck bruising.

CV: Tachycardic, regular rhythm.

Pulm: CTA B/L. No chest wall/rib deformity.

Abd: Diffuse bruising/withdrawal to pain all over the abdomen. Hypoactive BS.

Rectal: Good tone, no blood.

Pelvic: RT HIP ABDUCTED AND EXTERNALLY ROTATED, unstable pelvis.

Extremities: Weak, thready pulses. No deformities. Abrasions all over her body. Withdraws to painful stimuli.

Skin: Diffuse abrasions, pallor.

Neuro: Moaning, GCS 8.

Vital#1 ER initial vitals (once placed on the monitors):

HR 160

BP 75/40

Temperature 37.0

O2 Sats (RA) 95%

RR 35

After initial evaluation AND START of ATLS:

MANAGEMENT:

First steps:

Place patient on monitors, pulse ox, BP cuff

Airway:

Support airway (patient should be intubated or bagging should be continued)

Breathing:

Ask for assessment of breath sounds

Circulation:

Obtain 2 IVs and/or IOs

Administer at least one normal saline bolus 20ml/kg using Broselow chart

Administer two units of unmatched blood given push-pull

Ask for initiation of massive transfusion protocol

Place patient in pelvic binder for stabilization

FAST performance preferred but optional.

If all of the above met, patient’s VS will go to vitals #2.

If only fluid provided, no/insufficient blood given, and/or no pelvic binder placed within 7 minutes, the patient will decompensate and we will use vitals #3. MONITOR SHOULD ALARM TO PROMPT BLINDFOLDED PATIENTS TO CHECK FOR PULSE OR VS CHANGES.

Vital #2

HR 140

BP 85/50

Temperature 37.0

O2 Sats (RA) 95%

RR Intubated

Circulation continued: May give up to 3 total 20ml/kg boluses of normal saline. Continue to give blood products at 2:1:1 ratio until case end (will not ret more than one set of all products). Go to Vitals #2.5 if giving appropriate blood product resuscitation

Vitals #2.5

HR 130

BP 90/55

Temperature 37.0

O2 Sats (RA) 95%

RR Intubated

Once noted that circulation is improving but still not stable, resident should note that patient needs to be taken to the OR. Trauma surgery team should arrive for immediate OR transfer after appropriate summarization of primary survey findings.

**Monitor should alarm to prompt team leaders to ask for or look at vitals and check for a pulse prior to Vitals #3**

Vital #3

HR 150

BP 60/p

Temperature 37.0

O2 Sats (RA) 85%

RR Intubated

At this point, case will be run as PEA. Staff to perform full 2 minutes of compressions and rhythm/pulse check, plus Epi or not following PALS guidelines THEN END THE CASE with ROSC

LABS:

CBC, BMP, LFT, INR/PTT: Will not return during case

ABO RH: A Negative

IMAGES: If imaging is requested, we will provide verbal descriptions of findings to leaders, they will not be required to read them themselves

CXR: No signs of pneumo-hemothorax; if requested after intubation, will mention that endotracheal tube is in the appropriate location above the carina

Lateral C-spine x-ray: Normal with no deformities or fractures appreciated

Pelvis: Widening and separation of pubic symphysis consistent with pelvic fracture

FAST U/S: Free fluid seen in Morrison’s pouch

CLINICAL PROGRESSION:

 EM team will immediately take over care from paramedics. They will determine her GCS score of 8 or less and immediately attempt to secure an airway or continue to support with bagging. IO and/or 2 large bore IVs should be placed with normal saline bolus 20ml/kg ordered immediately. Ask for 2 units of trauma blood and activate massive transfusion protocol at either 1:1:1 or 2:1:1 ratio.

They should find the unstable pelvis, abdominal wall bruising, and additional suspected intra-abdominal hemorrhage (via positive FAST exam, although unnecessary if patient goes straight to OR due to suspected pelvic source of hemorrhage). A sheet should be tied around the pelvis. Patient will need aggressive fluid/blood resuscitation. Team will ask for labs. If US requested they will be described findings of fluid collection in Morrison’s pouch.

TRANSITION: Once the patient has had breathing supported, has been given fluids/blood, and massive transfusion has been activated, and trauma surgery has been contacted, patient will need for transport to the operating room (OR) for definitive management.

Primary objectives

a.      Communication skills

b.     Closed-loop communication

c.      Basic pediatric trauma management

d.     Identifying severe head injury which could affect spontaneous breathing

e.      Supporting breathing via BVM or securing definitive airway early

f.      Identifying ongoing hemorrhage

g.     Appropriate hemorrhage management (timely NS Bolus x2-3, type and screen, pelvic binder, blood transfusions, FAST, etc.)

h.     Activating massive transfusion protocol at appropriate ratio (2:1:1 or 1:1:1)

i.       Transferring unstable patient to OR for further management

Critical Actions

Place patient on monitors (+/- pulse ox and BP cuff)

Support breathing (via either definitive airway or bagging)

Obtain 2 large bore IVs or I/Os

Give patient normal saline bolus

Give patient trauma blood

Order additional blood via massive transfusion protocol

Use binder for control of the pelvic hemorrhage

Call surgical team and transfer to OR for definitive management
